# Suicidality, Bullying and Other Conduct and Mental Health Correlates of Traumatic Brain Injury in Adolescents

**DOI:** 10.1371/journal.pone.0094936

**Published:** 2014-04-15

**Authors:** Gabriela Ilie, Robert E. Mann, Angela Boak, Edward M. Adlaf, Hayley Hamilton, Mark Asbridge, Jürgen Rehm, Michael D. Cusimano

**Affiliations:** 1 Division of Neurosurgery and Injury Prevention Research Office, St. Michael's Hospital, Toronto, Ontario, Canada; 2 Social and Epidemiological Research, Centre for Addiction and Mental Health, Toronto, Ontario, Canada; 3 Dalla Lana School of Public Health, University of Toronto, Toronto, Ontario, Canada; 4 Department of Community Health and Epidemiology and Department of Emergency Medicine, Dalhousie University, Halifax, Nova Scotia, Canada; University of California, San Francisco, United States of America

## Abstract

**Objective:**

Our knowledge on the adverse correlates of traumatic brain injuries (TBI), including non-hospitalized cases, among adolescents is limited to case studies. We report lifetime TBI and adverse mental health and conduct behaviours associated with TBI among adolescents from a population-based sample in Ontario.

**Method and Findings:**

Data were derived from 4,685 surveys administered to adolescents in grades 7 through 12 as part of the 2011 population-based cross-sectional Ontario Student Drug Use and Health Survey (OSDUHS). Lifetime TBI was defined as head injury that resulted in being unconscious for at least 5 minutes or being retained in the hospital for at least one night, and was reported by 19.5% (95%CI:17.3,21.9) of students. When holding constant sex, grade, and complex sample design, students with TBI had significantly greater odds of reporting elevated psychological distress (AOR = 1.52), attempting suicide (AOR = 3.39), seeking counselling through a crisis help-line (AOR = 2.10), and being prescribed medication for anxiety, depression, or both (AOR = 2.45). Moreover, students with TBI had higher odds of being victimized through bullying at school (AOR = 1.70), being cyber-bullied (AOR = 2.05), and being threatened with a weapon at school (AOR = 2.90), compared with students who did not report TBI. Students with TBI also had higher odds of victimizing others and engaging in numerous violent as well as nonviolent conduct behaviours.

**Conclusions:**

Significant associations between TBI and adverse internalizing and externalizing behaviours were found in this large population-based study of adolescents. Those who reported lifetime TBI were at a high risk for experiencing mental and physical health harms in the past year than peers who never had a head injury. Primary physicians should be vigilant and screen for potential mental heath and behavioural harms in adolescent patients with TBI. Efforts to prevent TBI during adolescence and intervene at an early stage may reduce injuries and comorbid problems in this age group.

## Introduction

In North America, more than half a million youth under the age of 18 experience a Traumatic Brain Injury (TBI) that requires hospital-based care each year [1]–[4]. In the US and Canada, 50% of all injuries that kill and disable youth involve a TBI, and less severe injuries most often occur during sport activities [5]–[6]. Studies with clinical samples show impairments associated with TBI that include short- and long-term burdens related to cognitive, emotional and social functioning [1], [4]–[8]. Every year in the United States direct (e.g., medical care and rehabilitation) and indirect (e.g., low productivity and loss of work) medical expenses related to TBI are estimated around $77 billion with a similar burden observed in Canada and other similar countries [9]. The public health importance of brain injuries is highlighted in two recent reports from the Centres for Disease Control and the National Academy of Sciences' Institute of Medicine, who have set as their objectives a reduction in nonfatal TBI in adolescents and continued support of research on key mechanisms [3], [8]. Brain injuries during adolescence are particularly concerning because during adolescence the brain is still developing [1], [5], [8]. Furthermore, there is growing evidence that people who have had one or more TBIs are at greater risk of future TBIs, and evidence that multiple brain injuries can result in lasting cognitive impairment, substance use, mental health and physical health harms [6]–[7], [10]–[15].

However, little is currently known about the epidemiology and risk factors associated with TBI among adolescents. Internalizing (e.g., suicide, depression, anxiety, cognitive, emotional and/or social impairments associated with the injury), and externalizing factors (e.g., using alcohol and drugs to cope with the effects of the injury, risk taking, and conduct problems) represent common reasons for medical referral post-TBI, yet population based data examining these relationships are lacking [2]. A recent systematic review of work published between 2007–2012 found important evidence on the relationship between suicidality and TBI, and highlights the need of population-based data to evaluate this and related mental health and behavioural problems associated with TBI [16].

Clinical studies suggest that antisocial behaviour, having fewer friends, aggressive tendencies, and an inability to tolerate frustration are common post-TBI consequences during childhood and adolescence [7], [10]–[11], and that symptoms that often persist into adulthood could lead to vocational failure [12]–[15]. For example, in a small cross-sectional investigation that compared 7–17 olds with moderate to severe TBI with age-matched youth who acquired orthopaedic injuries, found correlates of poor social cognition (below age expected social relations with peers, deficient social cognitive skills) at three months after the injury [7]. Parents whose children (9 to 15 years old) had a TBI also reported that their children had greater peer relationship difficulties and also emotional distress, relative to parents whose children never acquired a TBI [11].

We report here an investigation of the adverse mental health and behavioural correlates of lifetime TBI in a large representative sample of adolescents in grades 7–12 in the province of Ontario, Canada. This investigation provides the first population-level data on this association in a large adolescent sample of TBI with important measures including suicidality and other mental health measures, bullying, and conduct behaviors.

## Method

Data for this study were derived from a subsample of the 2011 cycle of the Ontario Student Drug Use and Health Survey (OSDUHS), a repeated cross-sectional probability survey of Ontario students enrolled in grades 7 through 12 (ages 11–20) in publicly funded schools, representing about 93% of the province's adolescent population. The sample, which projects to nearly 1 million students, excludes private, military and institutional schools, and special education, English as a second language and low enrolment classes. Students completed anonymous, self-administered pen-paper questionnaires in their classrooms. The 2011 OSDUHS employs a stratified (region and school type [elementary, secondary]), two-stage (school, class) cluster sample design. Within each strata, schools were selected with probability-proportional-to-size, and within selected schools, classes were selected with equal probability. Questionnaires were administered in class between November 2010 and June 2011. The final sample consisted of 9,288 students drawn from 181 schools and 573 classes. The class participation rate was 62% and data did not present evidence of appreciable nonresponse bias [17]. A complete description of the protocol, sample design, survey's questions and their validation, by forms, including those we report here, and limitations is available on-line [17]. The study was approved by the Research Ethics Committees of the Centre for Addiction and Mental Health, St. Michael's Hospital, the participating Public and Catholic school boards, and York University who administered the surveys. All participants provided their own consent in addition to parental signed consent. One of the two randomly-distributed questionnaire forms employed in OSDUHS contained the mental health, suicide, bullying and conduct behaviour items, which we report here, and were answered by a subsample of 4816 students.

### Measures

#### Traumatic brain injury (TBI)

Lifetime TBI was assessed through the following question: “We are interested in any head injuries that resulted in you being unconscious (knocked out) for at least 5 minutes, or you had to stay in the hospital for at least 1 night because of it. Did you have this type of head injury in your life?” Responses included (1) Yes, I’ve had a head injury like this in the last 12 months, (2) Yes, I’ve had a head injury like this in my life, but not in the last 12 months, or (3) No, I’ve never had a head injury like this in my life.” The minimum of one overnight hospitalization due to symptoms associated with the head injury, or a minimum of 5 minutes loss of consciousness criteria are used in several classification systems including DSM-IV [18]–[22]. For analysis, responses 1 and 2 were combined to represent lifetime prevalence and were binary coded (1,1,0). This question, with an item response of 98%, is similar to those used in recent studies of self-reported TBI involving adults, although our study is the first general population survey involving adolescents [23]–[24]. Although we have no external data to validate our TBI measure, we do have concurrent items that provide correlational evidence of validity. One such item is any medically-treated injuries experienced in the past year. When correlating these items, a significantly positive relationship emerged (Cramer's V 0.21, P<.001). This correlation was both positive and significant, as expected. Moreover, we expect a modest correlation since the criterion includes injuries not restricted to TBI. To assess potential nonresponse bias, we compared high-participating classes (with 70% or more of students in the class participating; n = 323 classes) to low-participating classes (less than 70% participating; n = 258 classes), and found no evidence of nonresponse bias (19.6 vs. 19.8, t579 = −0.189, P = 0.850).

#### Mental/Emotional Health

The 12-item version of the General Health Questionnaire (GHQ12), a screening instrument was used to detect current elevated psychological distress [25]. The GHQ12 assesses depressed mood, anxiety, and social dysfunction. A cut score of three or more on the binary-scored GHQ12 is considered the validated threshold identifying someone experiencing elevated psychological distress [26]. Cronbach's reliability coefficient (α) for these 12 items in this sample is 0.89. The GHQ12 has been shown to be a valid screener among adolescents [17], [26].

### Suicidality

The suicide ideation and attempt questions asked: “In the last 12 months, did you ever seriously consider attempting suicide? In the last 12 months, did you ever actually attempt suicide?” Response options to both questions were yes or no. Both questions are from the Centre for Disease Control's Youth Risk Behaviour Survey (YRBS) and have demonstrated good reliability and validity among students [17], [27].

#### Seeking counselling through a crisis help-line/website

Students were asked: “In the last 12 months, have you phoned a telephone crisis helpline or gone on a website (such as “KidsHelpPhone.ca”) because you needed to talk to a counsellor about a problem?” Response options to this question were (1) yes, I phoned a helpline only; (2) yes, I posted a question on a website only; (3) yes, I phoned a helpline and posted a question on a website; (4) no. To ensure adequate cases, options 1,2 and 3 were combined.

#### Being prescribed medication for anxiety, depression or both

Students were also asked: In the last 12 months, have you been prescribed medicine to treat anxiety or depression? Response options to this question were (1) yes, anxiety only; (2) yes, depression only; (3) yes, both; (4) no. Responses 1, 2 and 3 were combined to insure adequate cases.

#### Bullying and conduct behaviours

Items measuring at school and cyber bullying assessed past 12 months occurrence, and were adapted from the World Health's Organization's Health Behaviour of School-aged Children (HBSC) study [4], [17]. Bullying was defined as repeatedly being teased by one or more people, being hurt or upset, or being left out of thing on purpose [17]. Students were asked if they were bullied at school since September. Response options included (1) was not bullied at school since September; (2) physical attacks (for example, beat you up, pushed or kicked you); (3) verbal attacks (for example, teased, threatened, spread rumours about you); (4) stole from you or damaged your things. Options 2 through 4 were combined to ensure sufficient cases. Cyber bullying was measured by asking, “In the last 12 months, how many times did other people bully or pick on you through the Internet?” Response options included (1) don’t use the internet; (2) never; (3) once; (4) 2 or 3 times; (5) 4 or more times. Options 1 and 2, and 3 through 5 were combined to represent having been cyber bullied. Students were also queried if they bullied other students since September. Response options included (1) did not bully other students since September; (2) physical attacks (for example, beat up, pushed or kicked them); (3) verbal attacks (for example, teased, threatened, spread rumours about them); (4) stole from them or damaged their things. Options 2 through 4 were combined.

#### Conduct behaviours

Conduct behaviours in the past 12 months were assessed by the following 11 items: being threatened/injured with a weapon (victim); taking the car without the owner's permission; damaging something on purpose that belonged to someone else; selling marijuana or hashish; stealing more than $50; setting fire; running away from home; breaking into locked building (not home); beating up or hurting someone (on purpose); carrying a weapon (e.g., gun/knife); getting into a fight at school at least once. Response options for all items included the following count scale: (1) never, (2) once, (3) 2 or 3 times, (4) 4 or 5 times, (5) 6 or 7 times, (6) 8 or 9 times, (7) 10 or 11 times, (8) 12 or more times. Options 2 through 8 were combined.

#### Analyses

To estimate variances from our stratified and clustered survey data, we employed Taylor Series Linearization (TSL) implemented in the Complex Sample module in SPSS V20.0. The estimation model was based on a design with 15 strata (region by school level) and 181 primary sampling units (schools). Binary logistic regressions, for each outcome, were estimated with lifetime TBI as the primary analytic predictor, with grade and sex as covariates, while accommodating the complex survey data. Two-way interactions between TBI x Sex and TBI x Grade were also examined in separate models. With listwise deletion of missing data the estimation sample was reduced from 4,816 to 4,685 students. The mean age of respondents was 15 (range: 11–20; SD = 1.81).

## Results

An estimated 19.5% (95% CI: 17.3, 21.9) of Ontario 7^th^ to 12^th^ graders acquired at least one TBI in their lifetime. The odds of acquiring a lifetime TBI were 47% higher among males than females (OR = 1.47). No statistically significant differences were found by grades. Students with TBI had significantly higher odds of reporting emotional and mental health issues (Table 1), being bullied, bullying others and displaying violent as well as non-violent conduct behaviours (see Table 2) than students without TBI.

**Table 1:** Percentage of students with and without a lifetime TBI reporting emotional and mental health indicators (N = 4,685), 2011 OSDUHS.

Indicator	No TBI % (95% CI) (n = 3803)	Lifetime TBI % (95% CI) (n = 882)	OR	95% CI	AOR	95% CI
Elevated psychological distress	32.4 (30.2–34.7)	39.2 (33.5–45.2)	1.35*	1.07–1.70	1.52**	1.19–1.94
Suicide ideation	9.2 (7.9–10.6)	15.2 (11.7–19.5)	1.78**	1.28–2.47	1.93***	1.42–2.63
Suicide attempt	2.0 (1.4–2.9)	5.9 (4.3–8.1)	3.05***	1.96–4.72	3.39***	2.15–5.35
Seeking counselling through a crisis help line	1.8 (1.2–2.7)	3.5 (2.3–5.4)	2.02*	1.13–3.60	2.10*	1.18–3.75
Was prescribed medicine to treat anxiety, depression or both	2.7 (1.8–4.1)	5.9 (3.4–9.8)	2.25*	1.00–5.06	2.45*	1.08–5.56

Notes: Unadjusted odds ratios (OR) and Adjusted Odds ratios (AOR) calculated in logistic regression models controlling for the effect of grade, sex and design; *** *P*<0.001,** *P*<0.01, * *P*<0.05, 2 tail-tests.

**Table 2:** Percentage of students with and without TBI reporting bullying and conduct behaviours (N = 4735), 2011 OSDUHS.

Indicator	No TBI % (95% CI) (n = 3803)	Lifetime TBI % (95% CI) (n = 882)	OR	95% CI	AOR	95% CI
Bullied at school (victim)	26.4 (24.2–28.7)	37.2 (29.4–45.8)	1.65**	1.17–2.32	1.70**	1.20–2.41
Bullied through internet (victim)	19.2 (17.6–21.0)	30.7 (23.5–30.0)	1.86**	1.29–2.69	2.05***	1.38–3.04
Threatened/ injured with a weapon (victim)	4.9 (3.6–6.5)	13.2 (9.5–17.9)	2.96***	1.88–4.67	2.90***	1.84–4.56
Bullied others	18.3 (15.4–21.6)	30.8 (21.1–42.6)	1.99**	1.29–3.07	2.05**	1.30–3.22
Took car for ride without the owner's permission	4.4 (3.2–6.1)	12.7 (8.8–17.9)	3.14***	1.73–5.69	3.47***	1.96–6.15
Damaged something on purpose that belonged to someone else	7.6 (6.3–9.1)	19.1 (15.4–23.4)	2.87***	1.99–4.15	2.88***	2.01–4.11
Sold marijuana or hashish	4.1 (2.7–6.2)	9.6 (6.1–14.8)	2.47***	1.38–4.42	2.58**	1.45–4.61
Theft more than 50$	2.7 (2.0–3.6)	8.3 (6.0–11.5)	3.25***	2.09–5.06	3.30***	2.12–5.13
Set fire	8.5 (7.1–10.3)	20 (14.9–26.3)	2.68***	1.93–3.71	2.67***	1.93–3.71
Run away from home	8.8 (6.9–11.1)	17.6 (13.7–22.3)	2.15	1.46–3.37	2.24***	1.48–3.38
Broken into locked building (not home)	3.6 (2.8–4.7)	7.4 (5.5–9.7)	2.11***	1.35–3.29	2.13***	1.38–3.28
Beat up or hurt anyone (on purpose)	7.1 (5.8–8.6)	14.7 (11.4–18.7)	2.27***	1.61–3.19	2.21***	1.57–3.12
Carried weapon(e.g., gun/knife)	3.4 (2.6–4.6)	9.3 (6.2–13.6)	2.86***	1.75–4.66	2.83***	1.72–4.65
Got in a fight at school at least once	9.4 (7.9–11.2)	21.6 (15.7–28.9)	2.64***	1.88–3.71	2.55***	1.81–3.58

Notes: Unadjusted odds ratios (OR) and Adjusted Odds ratios (AOR) calculated in logistic regression models controlling for the effect of grade, sex and design; *** *P*<0.001,** *P*<0.01, 2 tail-tests.

### Mental Health and Suicidality

Table 1 presents the regression analyses using TBI as the predictor while adjusting for sex, grade and survey design. Among students with TBI reported elevated psychological distress, suicide ideation, suicide attempt, and suicide ideation were higher compared to the non-TBI students, when factoring the influence of sex and grade, and the complex survey data. When controlling for emotional distress in addition to gender, grade and design, the odds of contemplating or attempting suicide were 1.68 (95% CI: 1.23, 2.30) and 2.89 (95% CI: 1.86, 4.49) times greater, respectively, and still statistically significant among TBI students compared to non-TBI students. The odds of seeking counseling through a telephone or web-based crisis help line in the past year as well as being prescribed anxiety/depression medication in the past year were significantly greater among TBI students compared with students who never acquired a TBI.

### Bullying and Conduct Behaviours

Table 2 presents the regression analyses results showing TBI as the predictor while adjusting for sex, grade and survey design. Being victimized during the current school year through bullying (at school, or via the internet) or being threatened at school with a weapon was significantly higher among brain injured than non-injured students, after adjusting for sex, grade and the survey data. When factoring the influence of sex and grade, the odds of bullying other students was also significantly higher among students with TBI than students without TBI.

Students with TBI were significantly more likely than students without TBI to report conduct behaviours. After controlling for the effects of sex, grade and survey data, the odds of reporting non-violent as well as violent conduct behaviours among students with TBI were statistically higher for all of the listed behaviours.

Comparison of the log-likelihood ratios for models with and without interaction terms between TBI x Sex and TBI x Grade did not show reliable improvements. Results indicate that the associations presented in Tables S1 and S2 did not vary between males and females, or by student grade.

## Discussion

Results indicate that 19.5% of adolescents in this population-based sample reported at least one brain injury in their lifetime that resulted in loss of consciousness for at least 5 minutes or at least one overnight hospitalization due to the symptoms associated with it. This estimate is closely related to the estimate of the larger sample from which these data were derived (20.2%) and slightly lower than the estimate of a large sample of Ontario adults surveyed the same year (16.8%) [6], [28]. Based on a report by the Centre for Disease Control in the US, the small difference between the adolescents and the adults sample may be explained by an increase (57%) in sports injuries among adolescents between 2001 and 2009 [29]. The report was based on hospital records alone and it showed that 6.5% of annual hospital sport related injuries among teens (ages 10 to 19) between 2001 and 2009 in the US were TBIs. This estimate is a little higher than the past 12 months estimate of TBIs among Ontario adolescents (5.6%) found in our larger sample reported in a recently published study [6]. Our estimate of lifetime reports of TBI is somewhat lower than self-reported lifetime prevalence estimates closer to 30% seen in some recent studies employing non-representative samples with a wider age range (25 or younger) [2], [29]. These differences are likely due to our age range (11 to 20) as well as our definition of TBI that captured mild to moderate levels of injury.

The results show strong relationships between self-reported TBI and mental health and behavioural problems in this large representative sample of adolescents. The odds of reporting mental health problems, such as elevated psychological distress, a higher use of anxiety/depression medication, increased suicidality, and seeking counselling through a telephone or web-based crisis help line, among students with a TBI were twice those of students without a TBI. These findings represent the first population-based evidence demonstrating the extent of the associations between TBI and poor mental health outcomes among adolescents, and point to the need for primary health care providers, as well as schools, to be aware of the potential comorbid conditions affecting the health and well-being of young people who have suffered TBI.

The results reported here show similar associations to those found in smaller scale studies that examined young offenders and adult populations [7], [14], [30]–[31]. TBI patients diagnosed with major depressive disorder are often found to exhibit comorbid aggressive behaviour, anxiety, significantly greater executive function impairment, as well as poor social functioning [14], [23]. Numerous adult studies also show significant associations of TBI with suicide behaviours, which may result from concomitant mental health difficulties and psychosocial disadvantages following TBI [16], [31]. In a recent systematic review of studies published between 2007–2012, the relationship between suicide and TBI was found to be robust, but no population-based studies were found for inclusion in the review [16]. Results presented here extend the literature by showing that even in a in a population based study of adolescents with lifetime TBI the odds of increased risk of suicide attempt or ideation as well as poor mental and psychological health compared with non-TBI adolescents are significantly higher.

Adolescents with lifetime TBI had twice the odds of being bullied at school or via the Internet and almost three times the odds of being threatened at school with a weapon compared with those without TBI. That TBI is associated with victimization experiences is yet another important indicator of the challenges experienced by adolescents post-TBI.^32^ The effects of victimization on adolescents, overall, can be traumatic, with victims of bullying reporting short- and long-term consequences such as anxiety, and depression which may persist into adulthood [31], [33]. Peer bullying and conduct behaviours could substantially exacerbate any emotional problems adolescents with TBI may be experiencing as a result of their injury (e.g., trouble focusing, paying attention to details), or might contribute to pre-existing self-esteem problems and interpersonal difficulties during this important developmental period.

These differences extend to externalizing behaviours such as bullying and other anti-social conduct. Adolescents who have experienced a lifetime TBI were also more likely, themselves, to be bullies and to engage in externalizing anti-social behaviours compared with students without TBI. Specifically, students with TBI reported increased past year involvement in behaviours such as damaging property, breaking and entering, taking a car for a drive without consent, selling marijuana or hashish, running away from home, setting fire, assaulting others, getting into a fight at school or carrying a weapon. Previous smaller scale studies have suggested that aggression may be a long-term outcome of paediatric [12]–[13] as well as adult TBI [15], [31], [34]. When untreated, these behavioural and emotional problems have been observed to persist later in life, manifesting in the form of elevated rates of social isolation, poor psychological adjustment, crime, and poor quality of life [10], [35], [36]. While some TBIs experienced by youth are unavoidable, many (e.g., those resulting from sports and recreational pursuits) are largely preventable [6]. Overall, our findings point to an urgent need for TBI prevention among this age group.

While this study makes significant contributions to the epidemiology of adolescent TBI, the findings here are bounded by limitations, such as the preclusion of causal inferences, possible bias related to self-reports, and underestimation due to the exclusion of institutionalized delinquent adolescents [17]. An important limitation is the lack of information about the temporal relationship between the report of lifetime TBI and the co-occurring adverse correlates identified here. It is not possible, in these data to establish whether these adverse correlates represent a coping mechanism to deal with the effects of TBI, or predisposing factors for adolescent TBI, or both. In a recent Australian study, more than 70% of a sample who had sustained severe TBI reported irritability or aggression that ranged from swearing to mild threats to violence that caused others physical injuries three years after sustaining the injury [35]. While other smaller scale studies show evidence of aggression post TBI [10], [15], [31], [34]–[36], some studies also point to the possibility that risk-taking behaviors and aggression can lead to injuries, including TBI [38]–[39].

Nevertheless, the results reported here are of significant interest as the first population-based adolescent data to demonstrate numerous internalizing and externalizing health associations with reports of lifetime TBI. These results show that preventable brain injuries and mental health and behavioural problems among teens continue to remain a blind spot in our culture. The potential for negative synergistic effects of combined TBI and mental health and substance use, including impact on academic performance, social and vocational failure, calls for a priority to be given to prevention and further research investigation. Targeted educative TBI prevention efforts in schools and communities are warranted, as well as directing parents, educators and primary care physicians and other health professionals to be vigilant in recognizing the high likelihood of adolescents with TBI to experience additional negative mental and physical health states and conduct problems.
